# Strengthening adherence to Anti Retroviral Therapy (ART) monitoring and support: operation research to identify barriers and facilitators in Nepal

**DOI:** 10.1186/s12913-015-0846-8

**Published:** 2015-05-05

**Authors:** Kiran Bam, Rajesh M Rajbhandari, Dibesh B Karmacharya, Sameer M Dixit

**Affiliations:** Center for Molecular Dynamics Nepal (CMDN), Thapathali, Kathmandu, Nepal

**Keywords:** Adherence, Anti-Retroviral Therapy (ART), Barriers, Facilitators, HIV, AIDS, Nepal

## Abstract

**Background:**

Anti Retroviral Therapy (ART) is the cornerstone for comprehensive health sector response to Human Immunodeficiency Virus (HIV) treatment, care and support. Adherence of at least 95% is needed to keep HIV under control, as per World Health Organization (WHO) guidelines. This study was aimed at identifying the overall adherence level of, and barriers and facilitators to adherence for patients taking ART in different clinics in all five development regions of Nepal.

**Methods:**

A descriptive cross-sectional study was conducted among ART clients receiving free ART from Government of Nepal ART clinics. A total of 435 clients taking ART from twelve ART clinics in different regions of Nepal, aged fifteen years and above were interviewed on one-and-one basis using questionnaires developed in reference to Adult AIDS Clinical Trial Group (AACTG) toolkit among them data from 404 were analyzed after cleaning. Data was entered and analyzed using Statistical Package for Social Sciences (SPSS) software where the P value of < 0.05 was accepted as being statistically significant.

**Results:**

The overall adherence in the last month (missed less than three pills total) was 94.8% (383 out of 404). The main barrier to ART adherence was the fear of side effects (among 61.9% of the non adherent population) which included dizziness (18.1%) and headaches (15.4%). The standard wristwatch (39%) was found to be the most useful aid in enabling timely consumption of medication. Educational status (P = 0.018), drug using habits (P = 0.039) and the conducive environment at ART clinics (P = 0.004) were significantly associated with ART adherence.

**Conclusion:**

Improving better adherence may require a more holistic approach to treatment regimen and adapting it to patient daily routines. This study identifies issues such as pill count for assessing adherence, better access to health care facilities by clients, better access to medication, as well as improved nutritional support issues for better adherence by the population in the future.

**Electronic supplementary material:**

The online version of this article (doi:10.1186/s12913-015-0846-8) contains supplementary material, which is available to authorized users.

## Background

Approximately 34 million people worldwide are currently living with Human Immunodeficiency Virus (HIV) and nearly 30 million people have died of Acquired Immune Deficiency Syndrome (AIDS)-related causes since the beginning of the epidemic [1]. Most People Living with HIV (PLHIV) or at risk for HIV do not have access to prevention, care, and treatment, and there is still no cure [2]. The progress of Anti Retro Viral (ARV) drugs has renovated HIV and AIDS to that of a chronic manageable disease. Earlier studies have reported improved quality of life of PLHIV on ART, reduced progression of the disease and declining mortality from the pandemic [3].

Nepal, with estimated 54,626 PLHIV is presently facing a concentrated epidemic with the adult (15–49 yo) HIV prevalence rate of 0.33% (2010). With support from the Global Fund to fight against AIDS, Tuberculosis and Malaria (GFATM), a national program providing free access to ART began in Nepal during 2004, and by the end of July 2010, a total of 6,754 clients had enrolled into treatment at the twenty five ART and ten sub ART clinics in Nepal [3]. In 2005, Nepal adopted a National ART Guideline, but even now, only 21% of those estimated to be in need are actually able to access ART services. There are estimated 17,000 people in need of ART in Nepal [4].

The standard ART service consists of the use of at least three ARV drugs to maximally suppress the HIV and to stop the disease progression [5]. An ART adherent person is one who does not miss more than three doses of ART treatment in a given time period [5].

Literature provides strong evidence that a non-adherent HIV patient on ART triple therapy is 3.87 times more likely to die than an adherent patient on the same therapy [6]. Additionally, benefits associated with ART compared with other less active forms of treatment are considerably reduced when the patient is non-adherent with the treatment [7]. Further, the risk of dying for an adherent patient on ART is nine times lower in comparison with the other types of treatment, this risk is only three times lower when the patient is non-adherent [7]. Adherence to ART is therefore clearly a critical factor in prolonging lives of PLHIV. The best response to ART is seen when adherence is 100%. Levels of adherence below 95% have been associated with poor suppression of HIV viral load and a lower increase in CD4 count [8]. Evidence suggests that greater than 95% adherence may be necessary to adequately suppress viral replication, produce a durable response and halt disease progression [9]. Previous studies [6,7] by different groups suggest that adherence to ART regimen among PLHIV is far from being satisfactory and better monitoring is required to achieve this. Both the PLHIV and the service providers have various reasons for ART regimen not being followed as prescribed.

Despite the availability of free ART services in Nepal, deaths due to AIDS still exist, and the prevalence of HIV among key affected populations still remains quite high [4]. In order for ART to work, clients have to stringently stick to the regime of medications. Failure to do so can undermine the effectiveness of the treatment and viral load can increase faster than it would have, otherwise. Furthermore, there is evidence from the field suggests that the second line ART regimen is being followed by some clients without completing the standard ART regimen. This brings in new queries regarding the efficacy of the ART on clients. Therefore, this is another issue that needs to be closely assessed as the ART adherence by clients is scrutinized. As the topographical difficulties, socio economic barriers coupled with the vertical nature of the current service delivery system, it is important to know the rate of adherence on ART by the clients. There is currently no country level ART adherence data. This study was thus initiated with the aim of a) identifying ways to best ensure that PLHIV follow ART regimen, b) measure the level of ART adherence at selected ART clinics, and c) identify gaps in ART adherence monitoring and support and to provide recommendations to scale up ART adherence. The expected results were to provide evidence what to address and/or promote to scale up the adherence and to contribute towards designing better directed and more culturally sensitive interventions to optimize the adherence rate to ART in Nepal. This would then serve as a resource for further research for developing new protocols for improvement in future.

## Methods

### Study design and site

**Figure 1:**
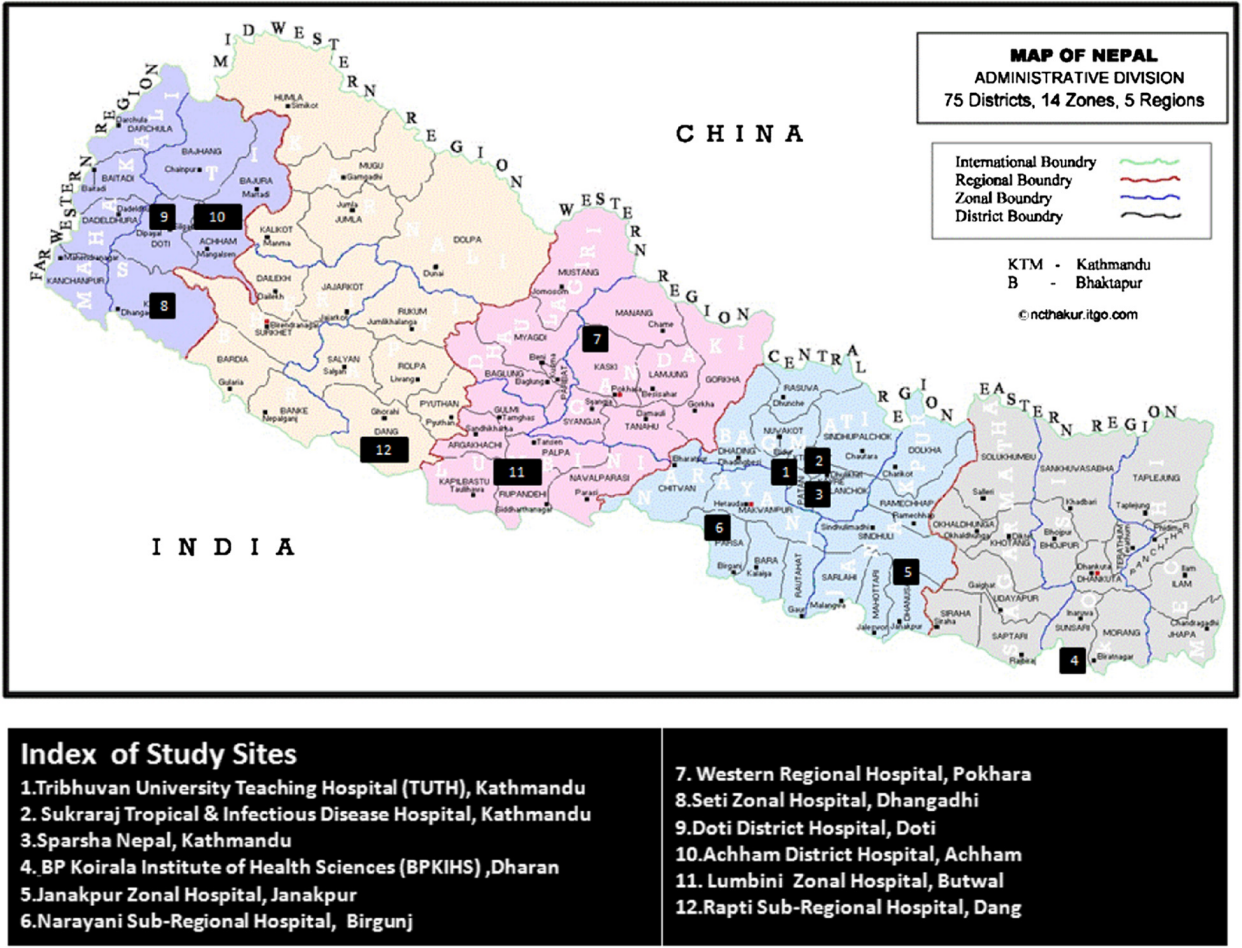
Study sites (map of Nepal adapted with permission from http://ncthakur.itgo.com/map04.htm).

A descriptive cross-sectional study was conducted at twelve ART sites (Figure 1) among then available twenty five ART sites of the Nepal, covering all the five development regions of the country (Table 1). A total of 435 clients on ART as per proportional sampling were randomly interviewed from July to November 2010.

**Table 1:** **Breakdown of selected ART sites by development region**

	**Far Western**	**Mid Western**	**Western**	**Central**	**Eastern**	**Total**
ART centers in the country	5	3	4	9	4	25
ART centers selected	3	1	2	5	1	12

### Study population

Clients on ART for at least six months above the age of fifteen years and who agreed to give informed consent were included in the study. For the participants of the study under the age of eighteen, informed consent was received from the parent or guardian. Participants who were too ill to be interviewed were excluded.

WHO sample size determination for health studies formula was used to determine the sample size of the study [10].The total sample size was determined to be 435 by taking 95% confidence interval, 84.0% level of ART adherence [11],5% margin of error, design effect of 2, Anticipating 5% non-response and incomplete interviews, and 80% power.

At 95% Confidence Interval (CI), with the allowable error of 5%. The sample size was calculated Sample Size $(n)=\frac{Z^2\times p\times \left(1-p\right)}{a^2}$

- p = 84.0%
- α = Allowable error = 0.05
- n = (1.96)²x0.84x0.16/ (0.05)²=206.5≃ 207.

A design effect of 2 was added to minimize the clustering effect as stated in other surveys sample size after including design effect-207x2=414. Anticipating chance of receiving incomplete interviews we then increased the final sample size by 5% which is now 435.

The multistage sampling technique was used to select the study participants. In the first stage, 12 ART clinics were randomly selected by the lottery method from 25 ART centers in different regions of Nepal (Table 1). In the second stage, the total of 435 samples was probability proportional to size allocated to each selected ART clinics. The participants were drawn from the PLHIV on ART list using simple random sampling.

### Study tools

Questionnaires looking into client socio-demographics, ART side effects as well as ART adherence information was adapted from Adult AIDS Clinical Trial Group (AACTG) which was translated into Nepali language, pre-tested and modified [12]. ART adherence was recorded as taking all ARV medications as prescribed by the physician. In this study, for adherence assessment, last one month self-reported adherence as mentioned in National ART guideline, 2005 was adopted [5]. Save the Children and National Center for AIDS and STD Control (NCASC)/ Ministry of Health and Population (MoHP) of Nepal were approached for evaluating the toolkit prior to finalizing the same. The toolkit was first field tested on a non-sample site, and after necessary adjustments, used for the study. The project was coordinated by the Center for Molecular Dynamics Nepal (CMDN), Kathmandu, Nepal.

### Data collection

Field research was managed by the Project Manager from CMDN and was jointly carried out by field researcher as well as other local field personnel. For minimizing recall bias, the answers were double-confirmed by asking the question more than once, allowing the participants time to remember the events.

### Operational definitions

#### Adherence

This is a self reported adherence to ART study in Nepal. This is the simplest method, and most extensively used. Patients are asked to report their own adherence – however, if used incorrectly it can be very inaccurate and will usually result in an overestimation of the level of adherence. Adherence was measured as per the mentioned criteria in National ART Guideline of 2005 [5] which is a) If less than three pills are missed during last one month (30 days), the person is >95% adherent; b) if 3–12 missed in the same period, 80-95% adherent; and c) if 12 pills missed, <80% adherent within one month of period [4]. The importance of strict adherence to ART cannot be overemphasized. Near perfect pill taking (>95% - [6]) is required to achieve maximal viral suppression – anything less than this leads rapidly to the development of viral resistance and hence, too much earlier treatment failure. WHO recommends at least 95% of adherence to ART to avoid the emergence of resistant strains of HIV. Therefore, we categorized the adherence level into two groups based on the above criteria- Adherent:Group A : >95% adherence; Non-Adherent:Groups B/C: <95% adherence.

**Non adherence:** Person missing more than three doses of treatment is considered as non-adherent.

**Infected duration:**Time since client first found to be infected (diagnosed) until the current time.

**Thought of leaving medication:** If clients felt like discontinuing ART.

**Satisfaction from ART clinic:** Perceived satisfaction from the ART clinic was measured. Respondents’ satisfaction with the health care providers was measured in the survey by asking participants whether the way health care providers treated them was ‘excellent’, ‘good’, ‘fair’, or ‘bad’.

### Data analysis

Data was entered and analyzed using the Statistical Package for Social Sciences (SPSS) version 17. Univariate and Bivariate analysis including chi-square tests, at 95% confidence interval were carried out to analyze the data.

### Possible errors and biases

Test subjects were clients that were selected from among those that visited the treatment centre. Thus selection bias against those not attending the treatment centre is possible. In order to limit this bias home visits to HIV positive persons on ART were carried out with the help of the Community Based Health Care (CHBC) workers.

Recall Bias may exist since the study is based on the self –report of the client. In order to minimize this bias the answers were reconfirmed by asking the question more than once, providing the clients enough time to remember events. Where possible, any other supporting information was also assessed to validate the answers. Local language was used to avoid the potential bias of misinterpretation of the questions.

### Limitations

The study is limited by its cross-sectional design, i.e. association cannot be interpreted as causal. The data management system was not electronic and thus some of the data was missed.

### Ethical approval

Ethical approval was obtained from the Ethical Review Board of Nepal Health Research Council, 2010 (NHRC/ MoHP/Government of Nepal) prior to initiation of study. Informed consent (written and verbal) was obtained from the each participant prior to interviews and data collection. Informed consent included information related to purpose of the study, potential risks and benefits of participating, procedures of maintaining confidentiality, right to not to participate in this study, as well as right to withdraw from interview at any point, were provided to the research subject.

## Results

A total of four hundred and thirty five (435) patients consented and participated in the study, out of which data from 404 records were analyzed. Thirty one were excluded for incompleteness. This represents a response rate of 92.9% of the number of patients eligible for the study. Among the thirty one patients who did not participate in the study, eight declined on confidential grounds, twelve did not give any reasons for the denying participation, and eleven did not done have initial CD4 counts.

In terms of age group of the participants, less than 35 years old population accounted for 44.4% of the total sample population. Males represented 52.0% of the study population (Table 2), and only 0.5% Third Gender representation. Among the clients interviewed, the majority were married (83.5%). Forty two participants (10.3%) were unmarried with a negligible number 13 (3.2%) that were either divorced or separated. The majority of the clients interviewed were of the ethnic *Janajatis* and *Chettris* groups (as per Nepal Census nomenclature). *Tharu* ethnicity accounted for the lowest representation amongst all ethnicities. The main religion observed amongst the clients was Hinduism (80.2%).

**Table 2:** **Socio demographic characteristics of participants**

**Socio-demographics**	**Number (n=404)**	**Percentage (%)**
**Gender**		
Male	210	52.0
Female	192	47.5
Third gender	2	0.5
**Age**		
<35 years	179	44.4
≥35 years	225	55.6
**Residence (according to development region)**		
Eastern	37	9.2
Central	117	29.0
Western	121	30.0
Far western	115	28.5
Mid western	14	3.5
**Caste (ethnicity)**		
Brahmin	47	11.6
Chettry	117	29.0
Tharu	9	2.2
Dalit	66	16.3
Janjati	144	35.6
Others	21	5.2
**Religion**		
Hindu	324	80.2
Buddhist	47	11.6
Muslim	10	2.5
Christian	20	4.9
Others	3	0.7
**Marital status**		
Married	337	83.5
Unmarried	42	10.3
Divorced	2	0.5
Separated	11	2.7
Others	12	3.0
**Education**		
Can’t read and write	105	26.0
Read and write	63	15.6
Primary	75	18.6
Lower secondary	65	16.1
Secondary	66	16.3
Higher secondary or higher	30	7.4
**Family income source**		
Agriculture	171	42.3
Service	96	23.8
Business	44	10.9
Labor	53	13.1
Others	40	9.9
**Family total income (NRs) /month#**		
<5000	189	46.7
5000-9999	127	31.4
≥10000	88	21.9
**Employment status**		
Employed	263	65.1
Unemployed	141	34.9
**Time to reach the ART clinic**		
<30 minutes	116	28.8
30 minutes-1 hour	108	26.8
1-2 hours	83	20.5
≥2 hours	97	24.0
**Smoking habit**		
No	322	79.6
Yes	82	20.4
Drug uses** habit		
No	374	92.5
Yes	30	7.5
**Disclosure of HIV status**		
Yes	318	78.7
No	86	21.3
**Infected duration**		
<1 year	82	20.3
1-4 years	227	56.1
≥5 years	95	23.5
**Treatment duration**		
<1 year	161	39.9
1-4 years	227	56.2
≥5 years	16	4.0

^**#**^1 Nepalese Rupees (NRs) = 0.0113770 USD (as of 3rd March 2013 from http://www.xe.com/currencyconverter/).

**Drug use includes marijuana, injecting drugs or other habitual drugs.

The majority of the ART clients (65.1%) were employed in various sectors at the time of the interviews. The majority of the ART clients were involved in the agricultural sector. The remainder were either in government service, business sector or worked as laborers. Their employment was also their major source of income. Most of the ART clients’ family income was less than NRs 5000 per month (46.7%). Among participants, 127 (31.4%) reported a family income between NRs 5000 and NRs 15000 (Table 2 defines the equivalent USD). In terms of educational level, 105 (26.0%) participant couldn’t read and write. Of the remainder (74.0%), almost equal number of participants had different levels of education (Table 2).

Most (93%) of the clients did not drink alcohol at all. Among the rest, there were both regular and rare drinkers of alcohol. In terms of smoking as a habit, a high percentage of participants said that they didn’t smoke (79.6%); rest (20.4%) said those smoked at various levels. Among the clients interviewed, 227 (56.2%) had initiated the ART regimen in the last 1 to 4 years.

### a. Status of last month ART adherence

**Figure 2:**
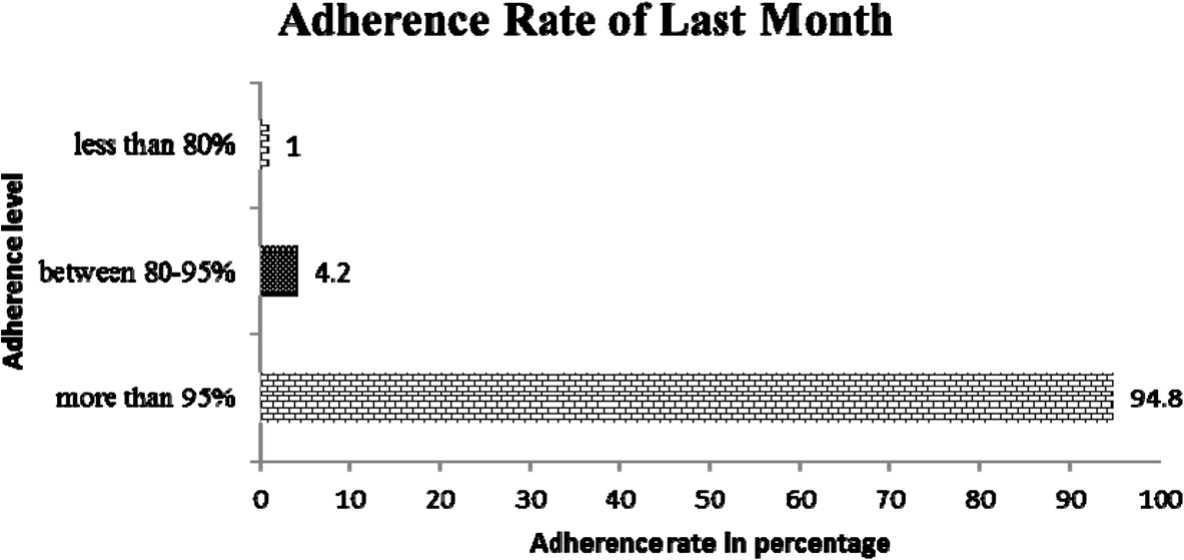
Clients' self reported adherence of last month.

Our study results have shown that the overall adherence in the population (>95%) was 94.8% (n = 383). The adherence as measured by the clients’ response to the number of pills s/he missed in the last month of the patient’s regimen. Among the rest, 4.2% were 80–95 % adherent while a small percentage (1.0%) showed less than 80% adherence (Figure 2).

### b. Factors associated with adherence to ART

Statistical analysis was carried out on key indicators playing a role in adherence amongst the adherent and non-adherent population. Most of the variables tested (for example: age, residence, ethnicity etc.) returned non-significant results (Table 3). However three parameters appeared to play a role in the adherence pattern of the clients. The most significant (P < 0.05) findings that affected ART adherence were a) level of education attained by participants (P = 0. 018), b) drug use habit (P = 0.039) and c) the environment of the ART clinic that the participants attended (P = 0.004).

**Table 3:** **Factors associated with adherence to ART**

**Variables**	**Adherent (≥95%)**		**Non Adherent (<95%)**		**P-value** ^**a**^
	**n = 383**	**%**	**n = 21**	**%**	
**Gender**					
Male	198	51.7	12	57.1	0.454
Female	183	47.8	9	42.9	
Third gender	2	0.5	0	0.0	
**Age**					
<35 years	172	45	7	33.3	0.772
≥35 years	211	55	14	66.7	
**Residence (according to region)**					
Eastern	34	8.9	3	14.3	0.116
Central	107	27.9	10	47.6	
Western	118	30.8	3	14.3	
Far western	110	28.7	5	23.8	
Mid western	14	3.7	0	0.0	
**Caste (ethnicity)**					
Brahmin	44	11.5	3	14.3	0.560
Chettry	112	29.2	5	23.8	
Tharu	7	1.8	2	9.5	
Dalit	63	16.5	3	14.3	
Janjati	136	35.5	8	38.1	
Others	21	5.5	0	0.0	
**Religion**					
Hindu	305	79.6	19	90.5	0.305
Buddhist	45	11.7	2	9.5	
Muslim	10	2.6	0	0.0	
Christian	20	5.3	0	0.0	
Others	3	0.8	0	0.0	
**Marital status**					
Married	319	83.3	18	85.7	0.772
Un married	39	10.2	3	14.3	
Divorced	2	0.5	0	0.0	
Separated	11	2.9	0	0.0	
Others	12	3.1	0	0.0	
**Education**					
Primary	67	17.5	8	38.1	0.018*
Lower secondary	60	15.7	5	23.8	
Secondary	63	16.4	3	14.3	
Higher secondary or higher	25	6.5	5	23.8	
Can’t read and write	105	27.4	0	0.0	
Read and write	63	16.4	0	0.0	
**Family income source**					
Agriculture	163	42.6	8	38.1	0.707
Service	88	23.0	8	38.1	
Business	44	11.5	0	0.0	
Labour	49	12.8	4	19.0	
Others	39	10.2	1	4.8	
**Family total income (NRs) /month** ^**#**^					
<5000	181	47.3	8	38.1	0.337
5000-9999	123	32.1	4	19.0	
>10000	79	20.6	9	42.9	
**Employment status**					
Employed	249	65.0	14	66.7	0.419
Unemployed	134	35.0	7	33.3	
**Time to reach ART clinic**					
<30 minutes	107	27.9	9	42.9	0.936
30 minutes −1 hour	105	27.4	3	14.3	
1-2 hours	80	20.9	3	14.3	
>2 hours	91	23.8	6	28.6	
**Alcohol habit**					
Never	356	93.0	19	90.5	
Once a month	10	2.6	0	0.0	
1-4 times a week	10	0.3	2	9.5	
Almost daily	4	1.0	0	0.0	
Daily	3	0.8	0	0.0	
**Smoking habit**					
No	307	80.2	15	71.4	0.448
Yes	76	19.8	6	28.6	
Drug uses** habit					
No	358	94.2	16	76.2	0.039*
Yes	25	5.8	5	23.8	
**Disclosure of HIV status**					
Yes	305	79.6	13	61.9	0.180
No	78	20.4	8	38.1	
**Infected duration**					
<1 year	78	20.4	4	19.0	0.283
1-4 years	218	56.9	9	42.9	
≥5 years	87	22.7	8	38.1	
**Treatment duration**					
<1 year	153	39.9	8	38.1	0.480
1-4 years	216	56.4	11	52.4	
≥5 years	14	3.7	2	9.5	
**Thought of leaving medication**					
No	341	89.0	16	76.2	0.180
Yes	42	11.0	5	23.8	
**Environment of ART clinic**					
Very good	220	57.4	11	52.4	0.004*
Good	62	16.2	3	14.3	
Satisfactory	1	0.3	2	9.5	
Not good	100	26.1	5	23.8	
**Medium of travel mode**					
Walking	48	12.5	2	9.5	0.898
Bus	258	67.4	15	71.4	
Own vehicle	21	5.5	0	0.0	
Rickshaw or Bicycle	49	12.8	4	19.0	
Others	7	1.8	0	0.0	
**Satisfaction from ART clinic**					
Yes	366	95.6	21	100	
No	17	4.4	0	0.0	

^**#**^1 Nepalese Rupees (NRs) = 0.0113770 USD (as of 3rd March 2013 from http://www.xe.com/currencyconverter/).

*Significant P values on 95% CI.

**drug use includes marijuana, injecting drug or other habitual drugs.

^a^P values were calculated from chi square test.

NRs: Nepalese Rupees.

ART:Anti Retroviral Therapy.

### Wristwatch as a major facilitator

Among those that reported more than 95% adherence in the last month (missed less than 3 pills), the leading facilitator in aiding timely consumption of the ARV medication was the common wristwatch worn by clients (39%). Family and friends support appeared to play an important role as well (38%) followed by radio (24%), timely availability of medicine (16%), and a prior collection of medicine other than the scheduled visit to ART (16%).

### Side effects as a major barrier

Among those clients reporting less than 95% adherence in the last month (missed more than three pills), large percentage 13(61.9%) mentioned that side effects were the major reason for avoiding the treatment followed by those stating being away from home 9 (42.9 %). Among others, lack of ART knowledge, forgetfulness, road closures (strikes), death of the spouse, transportation problems and no access to medications were causes for non adherence to ART (Table 4). Some of the side effects claimed by the participants as a result of ART were: dizziness (18.1%), headache (15.4%) and vomiting (13.8%).

**Table 4:** Reason for non adherence to ART(n = 21)*

**Reasons**	**n (% )**
Avoid side effects	13(61.9)
Away from home	9(42.9)
Lack of ART knowledge	9(42.9)
Afraid of taking medication	9(42.9)
Forgot to take medication	9(42.9)
No access to watch	7(33.3)
Road closures (strikes)	7(33.3)
Death of husband/spouse	7(33.3)
Transportation problems	7(33.3)
No access to medication	7(33.3)

*multiple response analysis was allowed.

## Discussions

Adherence to ART regimen is by no means easy. Yet it is a requirement to increase the longevity of PLHIV. There are various difficulties a patient, already weakened by the suppressed immune system would face in continuing the daily ART routine. This study has attempted to outline the major difficulties faced by clients in developing country such as Nepal, as per information from national guidelines, WHO, as well as other global HIV bodies. These major areas were used as parameters to assess the barriers to effective implementation of national ART guidelines at the community level.

Our study revealed an overall adherence (>95%) of 94.8%. This is higher than a previously conducted similar study focusing on far western HIV population that showed 84% [11]. Similar studies carried out elsewhere have shown far lower >95% adherence results. It is higher than the studies conducted in south Asian developing countries such as India, China and Thailand [13-15]. In addition to those, adherence percentages of 43.2% in Kenya [16] and 62.5% in South Africa respectively [17] have been reported. However, Lower adherence at 48% has been reported in Ethiopia [18]. In that regard, the adherence observed in our study can be considered relatively satisfactory as 95% are adherent to the treatment. This can be attributed to increased awareness of ART in the community and the benefits provided therewith, mobilization of the CHBC, family support and self-report of the ART adherence.

The simple wristwatch of clients appeared helpful in supporting ART adherence in the clients. This watch was instrumental in helping clients keep tab of time to take their medication. Similar findings have been reported in Ethiopia, Botswana, Tanzania and Uganda [19-21] where memory aids have been used for reminder regarding medication consumption. In a previous study carried in Nepal to assess ART adherence, the watch was used in a similar fashion [11].

Furthermore, the findings also showed that the majority of ART clients did not smoke, and they did not consume alcohol. This may reflect a sense of responsibility by the clients towards the treatment, and enhanced awareness about the importance of ART and its relation to HIV therapy through CHBC’s. Worldwide, alcohol use has been documented as a prime barrier to treatment adherence [22].

This indeed appears to be true for the good adherence as the clients were very aware of the ART since most have had pre- and post- ART counseling. This, and the fact that in the majority of the cases they were satisfied with the services provided at the ART sites (in terms of waiting time, confidentiality, communication channels among others) appears to have played a role in helping the clients maintain adherence to their medication regimen. Consistent with other research of the influence of patient-provider relationships on ART adherence [23,24], this study found that the perceived quality of information from health care providers was positively associated with adherence.

Numerous studies have reported that experience of side effects against prescribed medication tends lead to non-adherence or cessation of intake [25-27]. In our study, high percentage of non adherent clients (61.9%) reported side effects as being the major cause for irregular adherence to ART. There are several reasons behind this; one of the reason is that ART was started when the CD4 count reached ≤ 200 cells/mm^3^ (as compared to WHO recommendation of ≤ 350 cells/mm^3^) which means treatment was started after the immune system was already weakened, thus leading to heightened side effects. Lower adherence is associated with experience of medication side effects [28]

Although being away from home is a possible reason for non-adherence as per literature [11], this does not appear to be the case in our study. This could primarily be due to the fact that social support groups are active in Nepal and are usually placed near residential areas. This enables timely intervention by workers to reach the client and provide advice on ART.

Our finding suggest that increase in education level (including secondary, higher secondary and higher) was not related to better adherence; lower level educational status showed better adherence. This contradicts to the finding of study conducted in Nigeria [29]. Participants with a history of various drug habits (e. g. Marijuana, injecting drug or other habitual drugs) have been linked with lower adherence level [30,31].

The majority of the participants in our study that had drug habits were unemployed, likely to be involved in illicit drug related transactions as source of funds for drug purchase. The positive environment of ART clinics appears to be directly related to good adherence – a finding similar to that found in a study from Africa [21].

This study did have certain field limitations in terms of accessing information as data source. The main limitations was that the pill count was not carried out while interviewing the participants. This was difficult to carry out as study sites were ART clinics where clients come once a month (every ART clinic offers in an average once month medicine) to refill medication. In most cases, those clients do not bring their remaining pills, if any. Further, the clients were unable to, in many instances, reveal their CD4 counts at the start of ART regimen. Further, this study relied on interviews with ART clients, and as such was based on self-reporting. Recall bias would always be possible in such instances.

## Conclusions

This study has identified some of the barriers to adherence in a developing country setting, and in particular, in a country with difficult geographical terrains and limited modes of transport. Distances between health care settings and homes, as well as means to get between those are some of the impediments identified by this study, for ART adherence.

The most significant finding was that education, drug use habits and the environment of ART clinics affected adherence to ART. Results from this study have clearly demonstrated that having a higher level of education was more likely to result in increased adherence as compared to basic education. Furthermore, the drug uses habit group appeared to have more difficulty maintaining adherence as compared to habit of none drug users. This finding clearly identifies the need to pay special attention to this key affected population regarding ART medication in Nepal. Also, having a positive opinion about the healthcare centers that provide medication as well as counseling appeared to positively influence ART adherence and should thus be promoted.

This type of study, if carried out at a national level would be expected to provide a more comprehensive information on national ART adherence and behavioral data. This study can also have a role in helping international community understand the difficulties of ART implementation in developing countries. Together, better plans and policies can be formulated to make ART adherence 100% all over the country and in other developing countries as well. Already, the adherence rate in Nepal far exceeds those observed in other developing countries in Africa. However, there is always room for improvement, and opportunities to save more lives.

While this study identified major gaps in ART adherence by clients, there are still areas for further studies to try to understand remaining gaps. A requirement for pill count at ART clinics could be an important mechanism to support adherence to the regimen. There also is a need for ART clinics to be decentralized- instead of current district headquarters implementing the treatment, health posts and sub health posts across the country should also be utilized to provide such services. There is also a need to implement cross-country coordination mechanisms between Nepal and neighboring India where Nepali migrant workers travel for work, whereby Nepali ART clients can access ART services at a place of employment in India.

This study also shows that there is a need to increase care homes, expand CHBC worker access to clients, and increase ART clinics in all 75 districts of Nepal in order to improve access to medication thereby facilitating adherence to ART. Further, improving currently implemented governmental nutritional support for ART clients, improving healthcare facilities so that the clients are more likely to visit and adhere to ART regimen, are some of the recommendations from the study findings, towards improving adherence in Nepal by clients on ART.
